# Supplementary material for: Predicting visual function by interpreting a neuronal wiring diagram
Source: Nature. 2024 Oct 2;634(8032):113–23. doi: 10.1038/s41586-024-07953-5 (PMC11446822; doi:10.1038/s41586-024-07953-5)
Supplement: Supplementary file 6 — CRF and ERF predictions for individual TmY4 and TmY9 cells. Analogous to Supplementary Data 3, but for TmY target types. Shown are the top four monosynaptic pathways, the strongest pathway passing through each of the top ten intermediary types (ranking from Extended Data Fig. 7), and the trisynaptic pathway Tm1–TmY–Dm3–TmY (see the section entitled Prediction of spatial normalization). [file 41586_2024_7953_MOESM6_ESM.zip › DataS4/TmY9qΓèÑ/720575940627012636.pdf]

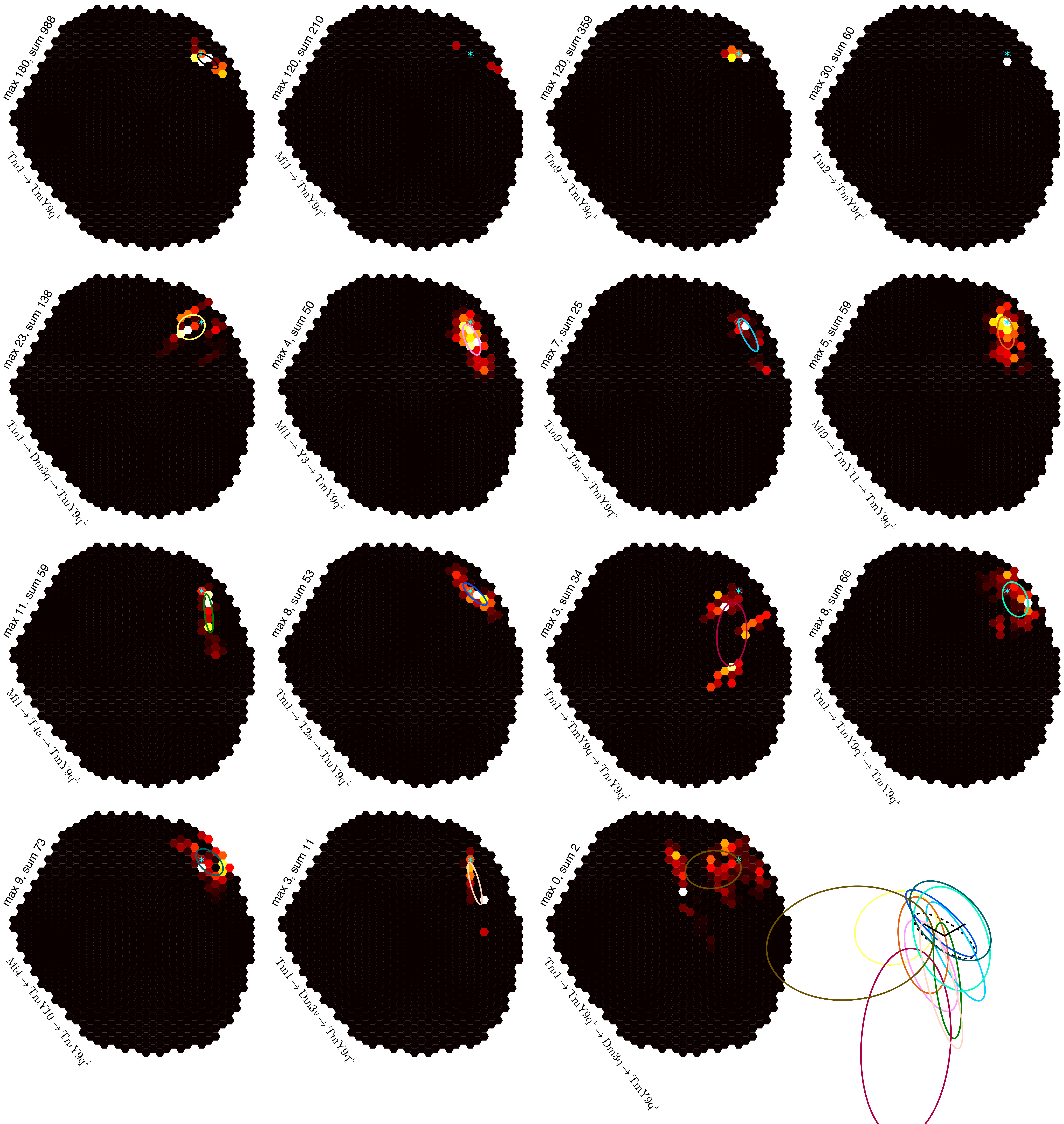
max 180, sum 988
Tm1 → TmY9q⊥
max 120, sum 210
Mi1 → TmY9q⊥
max 120, sum 359
Tm9 → TmY9q⊥
max 30, sum 60
Tm2 → TmY9q⊥
max 23, sum 138
Tm1 → Dm3q → TmY9q⊥
max 4, sum 50
Mi1 → Y3 → TmY9q⊥
max 7, sum 25
Tm9 → T5a → TmY9q⊥
max 5, sum 59
Mi9 → TmY11 → TmY9q⊥
max 11, sum 59
Mi1 → T4a → TmY9q⊥
max 8, sum 53
Tm1 → T2a → TmY9q⊥
max 3, sum 34
Tm1 → TmY9q → TmY9q⊥
max 8, sum 66
Tm1 → TmY9q⊥ → TmY9q⊥
max 9, sum 73
Mi4 → TmY10 → TmY9q⊥
max 3, sum 11
Tm1 → Dm3v → TmY9q⊥
max 0, sum 2
Tm1 → TmY9q⊥ → Dm3q → TmY9q⊥
